# Recent progress on DNA methylation in psoriasis

**DOI:** 10.3389/fgene.2025.1681666

**Published:** 2025-10-01

**Authors:** Zhaoping Lin, Xiaoting Wu, Xuqin Hong, Dongying Luo, Hui Li, Fei Ma, Cong Huang, Bo Yu, Jing Gao, Changbing Shen

**Affiliations:** ^1^ Department of Dermatology, Peking University Shenzhen Hospital, Shenzhen, Guangdong, China; ^2^ Shenzhen Key Laboratory for Translational Medicine of Dermatology, Institute of Dermatology, Shenzhen Peking University - The Hong Kong University of Science and Technology Medical Center, Shenzhen, Guangdong, China; ^3^ Department of Dermatology, PKU-Shenzhen Clinical Institute of Shantou University Medical College, Shenzhen, Guangdong, China; ^4^ Department of Dermatology and Venereology, The Second Affiliated Hospital of Anhui Medical University, Hefei, Anhui, China; ^5^ Joint Laboratory for Plasma Clinical Applications, The Second Affiliated Hospital of Anhui Medical University, Hefei, Anhui, China; ^6^ Department of General Practice, The Second People’s Hospital of Futian District, Shenzhen, Guangdong, China; ^7^ Department of Anesthesiology, The Second Clinical Medical School of Anhui Medical University, Hefei, Anhui, China

**Keywords:** psoriasis, epigenetics, DNA methylation, pathogenesis, progress

## Abstract

Psoriasis is a chronic, recurrent, inflammatory disease that is affected by genetic, immunological, epigenetic, and environmental factors. With the development of biotechnology, research on the pathogenesis of psoriasis has deeply focused on the field of epigenetics, and great progress has been made. Epigenetics is the study of heritable changes in gene expression or cell phenotypes without altering the DNA sequence. DNA methylation (DNAm) alterations are the most common epigenetic phenomena and are widely studied. Many studies have shown that DNAm plays a key role in the pathogenesis of psoriasis, and some differentially methylated sites may be potential targets for the treatment of psoriasis. Here, we review and summarize the recent progress on DNAm in psoriasis.

## Introduction

Psoriasis is a chronic inflammatory disease that poses a huge economic and psychological burden on both individuals and society (Griffiths et al., 2021). The prevalence of psoriasis across different ethnic populations ranges from 0% to 2.1% in children and 0.91%–8.5% in adults (Parisi et al., 2013). The pathogenesis of psoriasis has not been fully clarified, and it is believed that the occurrence of psoriasis is due to genetic (Sheng et al., 2014; Yin et al., 2015; Zuo et al., 2015; Zhou et al., 2016a), immunological (Huang et al., 2021; Huang et al., 2025), epigenetic (Zhou et al., 2016b; Zhou et al., 2016c), and environmental factors (Griffiths et al., 2021). With the development of biotechnology, research on psoriasis pathogenesis has focused on epigenetics. Epigenetics is the study of heritable changes in gene expression or cell phenotypes without altering the DNA sequence. Many epigenetic phenomena, such as DNA methylation (DNAm), genomic imprinting, maternal effects, gene silencing, nucleolar dominance, dormant transposon activation, and RNA editing, participate in the occurrence and development of diseases. Previous studies have shown evidence of anatomical location-dependent DNAm patterns in psoriatic lesions (Wu et al., 2019), an association between biological age and DNAm age (Shen et al., 2018), consensus clustering based on DNAm data (Zhou et al., 2018), DNAm of some genes mediating risk variation for psoriasis (Zhou et al., 2016c), and DNAm loci for psoriasis identified by epigenome-wide association analysis (Zhou et al., 2016b). In recent years, an increasing number of researchers have focused on the epigenetic pathogenesis of psoriasis and achieved many meaningful results. Here, we review and summarize the recent progress on DNAm in psoriasis.

## Materials and methods

We performed a literature search in PubMed using the search terms “(Psoriasis) AND (DNA methylation)”, as of 3 September 2025, a total of 164 articles were retrieved. Since the aim of this article is to summarize and review the latest progress of DNAm in psoriasis, we mainly focus on the literature published in the past 5 years, and we carefully read the full texts and summarized the research findings.

## Role of DNA methylation in the pathogenesis of psoriasis

### DNA methylation in psoriasiform mouse models

**FIGURE 1:**
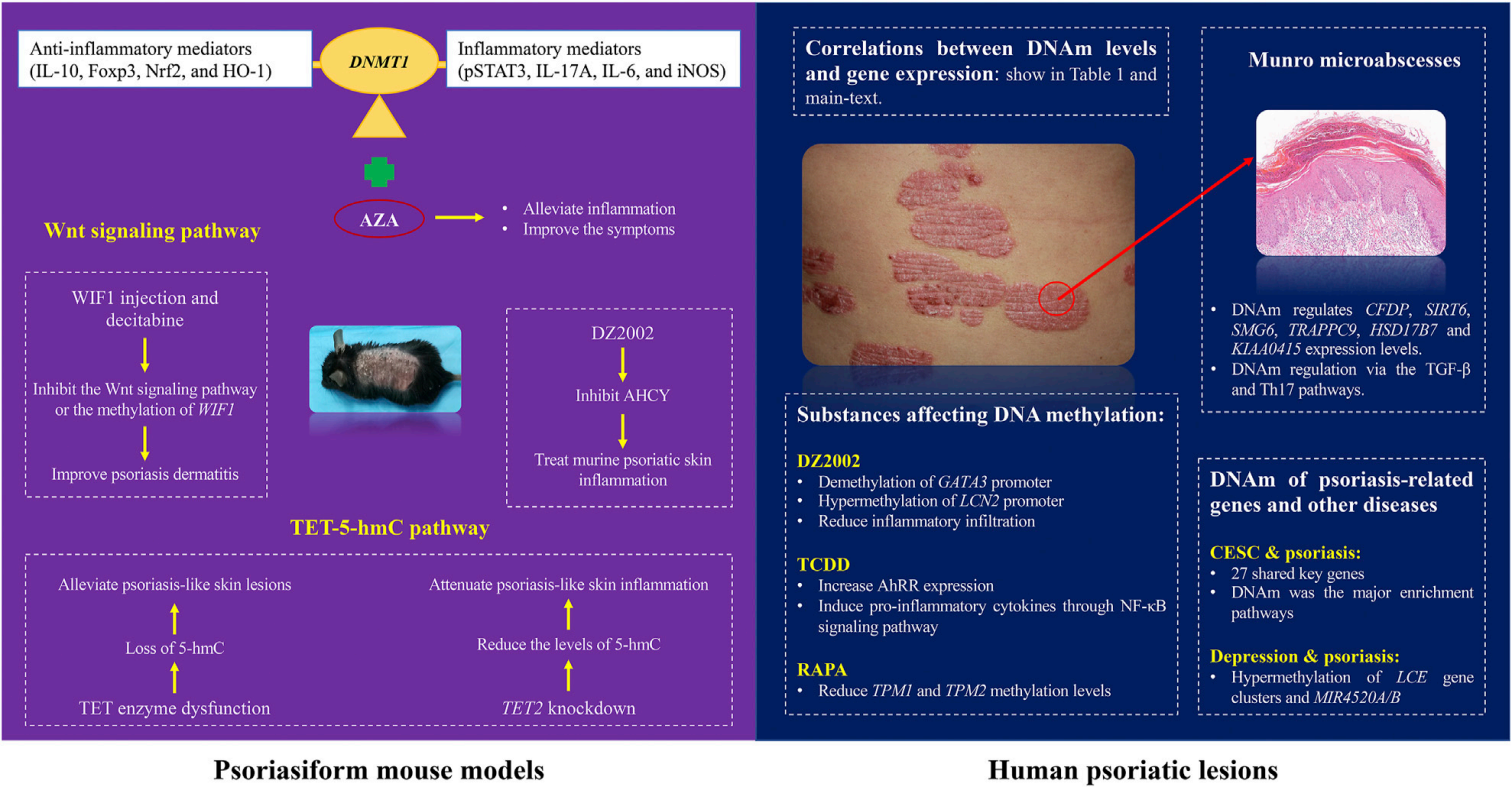
Role of DNAm in the pathogenesis of psoriasis.

The role of DNAm in the pathogenesis and treatment of psoriasis has been explored using animal models (Figure 1). Previous studies have shown that DNAm regulation relies on methyltransferases (DNMT) 1 (Greenberg and Bourc’his, 2019). In imiquimod (IMQ)-induced mouse models, the expression of *DNMT1* and DNAm was increased in a mouse model of di (2-ethylhexyl) phthalate (DEHP) exposure (Alfardan et al., 2024). It is related to the increase in inflammatory mediators (pSTAT3, IL-17A, IL-6, and iNOS) and downregulation of anti-inflammatory mediators (IL-10, Foxp3, Nrf2, and HO-1), and the DNMT inhibitor, 5-aza-2′-deoxycytidine (AZA), can alleviate inflammation and improve the psoriasis-like symptoms in mice.

DNAm also plays a critical role in psoriasis through the Wnt signaling pathway. Verma et al. (2023) found that differentially methylated miRNA-encoding genes in psoriatic epidermis were closely related to the Wnt signaling pathway. Liu et al. (2023a) found that the expression of Wnt inhibitor 1 (*WIF1*) was reduced at both the mRNA and protein levels in psoriatic lesions and that the *WIF1* promoter region was hypermethylated. They further found that recombinant WIF1 injection and decitabine, improved psoriasis dermatitis in an IMQ-induced mouse model. These findings suggest that inhibiting the Wnt signaling pathway or the methylation of *WIF1* may serve as potential therapeutic strategy for psoriasis.

There is increasing evidence of the importance of epigenetic modifications in psoriasis progression. Ten-eleven translocation-2 (TET2), an enzyme that catalyzes the conversion of 5-methylcytosine (5-mC) to 5-hydroxymethylcytosine (5-hmC) and promotes DNA demethylation, was found to be highly expressed in IMQ-induced psoriasis-like skin lesions in mice (Wang et al., 2018). In a mouse model, Wang et al. demonstrated that *TET2* knockdown significantly reduced the levels of 5-hmC in the lesional skin of mice, decreased the expression of pro-inflammatory cytokines (IL-17A, IL-17F, and interferon-γ) and the chemokine *CXCL1*, and alleviated psoriasis-like skin inflammation. In human and experimentally-induced psoriatic lesions, the loss of TET expression and the decrease in 5-hmC levels could be related to stem cell dysregulation, which is consistent with the epidermal characteristics of psoriasis (Li et al., 2020; Van Der Fits et al., 2009). A study shows that Ascorbic acid derivative DDH-1 alleviate the psoriasis-like lesions in mice, which may suppress NF-kB signaling by inducing PPAR-a and PPAR-y expression and reduce inflammatory cytokine expression (Li et al., 2020; Gao and Si, 2018; Kitahata et al., 2018). These studies reflect the complexity of the epigenetic regulatory mechanism of psoriasis, suggesting that epigenetic modifiers in the TET-5-hmC pathway may be potential therapeutic targets for psoriasis.

### DNA methylation regulates the expression of key genes in psoriasis

A number of studies have focused on the relationship between DNAm and genes related to psoriasis (Table 1). Negative correlations have been reported between DNAm levels and gene expression (Chandra et al., 2018). Significant causal relationships between DNAm, gene expression, and protein levels, and psoriasis risk were determined by summary-data-based Mendelian randomization (Guo et al., 2025). DNAm at certain sites (negative regulation: cg26804944 and cg02705573; positive regulation: cg00172967, cg00294382, and cg24773560) were associated with the expression of *RP11-977G19.11*. Liu et al. (2023b) identified differentially methylated and expressed genes (DMEGs) in the psoriatic epidermis and screened six hub genes (*GZMB*, *CRIP1*, *S100A12*, *ISG15*, *CRABP2* and *VNN1*), whose transcription levels showed a significant correlation with psoriasis area and severity index (PASI) scores and immune infiltration, suggesting that epidermis-specific hub genes are potential biomarkers for judging the severity of psoriasis. HLA-Cw*0602 has long been established as one of the most important genetic biomarkers for psoriasis. Tang et al. (2020) found that HLA-Cw*0602 carriers of psoriasis exhibited significant methylation differences compared to non-carriers, including low DNAm of genes such as *KLF7*, *PIP5K1A*, *BTBD10*, and high DNAm of *GOLGA2P5*. These differences are mostly located in the gene body and CpG islands, which are involved in psoriasis pathogenesis through multiple pathways.

**TABLE 1:** Recent research findings on the regulation of gene expression through DNAm.

Published year	Population	Main findings	References
2018	Chinese	• In involved psoriatic skin tissues (PP) compared to normal skin tissues (NN), 1,460 genes were detected to be significantly differentially expressed (565 genes upregulated and 895 genes downregulated). A noted negative correlation between DNAm and expression levels of 485 differentially expressed genes (DEGs). • In the comparison of PP and uninvolved psoriatic skin tissues (PN), 818 hypermethylated genes with downregulated expression and 522 hypomethylated genes with upregulated expression. The expression levels of 758 DEGs were negatively correlated with DNAm. • 290 overlapped DEGs in PP compared to NN and in PP compared to PN, the top 20 dysregulated genes are *SERPINB3*, *C10orf99*, *OASL*, *OAS2*, *MX1*, *ZC3H12A*, *LAMP3*, *IFIT3*, *SERPINB1*, *IRF7*, *C1QTNF7*, *IL34*, *GPIHBP1*, *HSD11B1*, *PLIN1*, *PHYHIP*, *PRR15L*, *HSPB7*, *PPP1R1B*, *GPD1*.	Tang et al. (2018)
2018	Indian	• Significant downregulation for hypermethylated genes (*SELENBP1* and *ZNF106*). • Significant upregulation for hypomethylated genes (*PTPN22* and *S100A9*).	Chandra et al. (2018)
2020	Chinese	• Significant methylation differences at 4,321 sites (811 hypomethylated sites and 3,510 hypermethylated sites) were detected. • The cg02607779 (*KLF7*), cg06936779 (*PIP5K1A*), cg03860400 (*BTBD10*) and cg26112390 (*GOLGA2P5*) were identified and validated to be the significant CpGs to different HLA-C*0602 groups.	Tang et al. (2020)
2021	Chinese	• 632 and 274 differentially accessible region-linked genes were significantly upregulated and downregulated, respectively in PP samples. Example: The methylation level of cg07195224 was strongly and negatively correlated with AIM2 mRNA expression.	Tang et al. (2021)
2021	Indian and Chinese	• 3,265 differentially methylated CpGs were identified (666 differentially methylated CpGs (DMCs) were hypomethylated, and 2,599 DMCs were hypermethylated). • 2,406 differentially methylated genes were identified (1,953 genes were hypermethylated and 569 genes were hypomethylated). • 1,538 differentially expressed-methylated genes were identified (1,188 hypermethylated genes, 350 hypomethylated genes).	Liu et al. (2021)
2022	Mixed	• 13 common genes were identified in the list of downregulated DEGs and hyper-differentially methylated region (DMR)-genes (hyperdownregulated genes): *TRIM2*, *HOXB3*, *TNXB*, *C1QTNF7*, *ESR1*, *CFL2*, *CCND1*, *DIXDC1*, *HLA-DQB2*, *PRLR*, *MACROD2*, *RORA*, and *ZSCAN18*. • 20 common genes in the list of upregulated DEGs and hypo-DMR-genes (hypoupregulated genes): *TAP1*, *S100A9*, *EPSTI1*, *GJB2*, *GRHL3*, *TTC22*, *SOX7*, *WNT5A*, *XAF1*, *GJB6*, *LAD1*, *POLR3G*, *KPNA2*, *E2F8*, *MX1*, *LTF*, *EPHX3*, *LGALS3BP*, *NUSAP1*, and *ESRP2*. • *GJB2* was the final identified hub gene.	Xing et al. (2022)
2022	Chinese	• DNAm at cg26853064, cg13663667, cg12946690, cg26429042, cg14779990, and cg09394666 were negatively correlated with the expression of *CFDP*, *SIRT6*, *SMG6*, *TRAPPC9*, *HSD17B7*, and *KIAA0415*, respectively.	Xu et al. (2022)
2023	Chinese	• 1,695 nonredundant differentially methylated and expressed genes (DMEGs) identified. • 693 genes were hypomethylated with upregulated expression and 1,002 genes were hypermethylated with downregulated expression.	Liu et al. (2023b)
2025	European	• DNAm at cg26804944 and cg02705573 were negatively associated with the expression of *RP11-977G19.11*. • DNAm at cg00172967, cg00294382, and cg24773560 were positively associated with the expression of *RP11-977G19.11*.	Guo et al. (2025)

Studies have compared the DNAm patterns of psoriatic skin (PP), uninvolved psoriatic skin (PN), and normal skin (NN), revealing the regulatory role of specific DNAm genes in psoriasis and their clinical value as biomarkers. Tang et al. (2018) conducted an integrative analysis to elucidate the biological pathways by which DNAm contributes to psoriasis pathogenesis. By comparing differentially methylated sites (DMSs) and differentially expressed genes (DEGs) among PP, PN, and NN, they found that upregulated genes were primarily enriched in T cell activation, type I interferon signaling pathway, and defense response to other organisms, with notably activated NOD-like receptor signaling pathways. Conversely, the downregulated genes were mainly enriched in the regulation of lipolysis in adipocytes, cGMP-PKG signaling pathway and Wnt signaling pathway. This study revealed that the biological pathways involved in psoriasis included enhanced innate immunity and reduced lipid biosynthesis. 20%–50% of patients with psoriasis are affected by metabolic syndrome, the psoriatic lesion contains more arachidonic acid metabolites, lipid metabolites can intensify oxidative stress and trigger inflammation, so that further affects the occurrence and development of psoriasis (Chen et al., 2023).

By performing assay for transposase-accessible chromatin using sequencing and multi-omics integration analysis of PP, PN, and NN, Tang et al. (2021) found that chromatin accessibility was significantly increased in psoriasis lesions, and genomic regions with differential accessibility were more hypomethylated, which affected several psoriasis susceptibility loci. These differentially accessible regions are rich in Fra-1 and/or activator protein-1 transcription factor DNA-binding motifs, which activate AIM2 inflammasomes and promote inflammatory responses in patients with psoriasis. *AIM2* is a susceptibility gene for psoriasis, and play important roles in the pathogenesis and treatment of psoriasis (Wang et al., 2022). A study on DNAm in peripheral blood samples from 41 patients with psoriasis and 30 healthy controls found that the overall DNAm level in patients with psoriasis was slightly higher than that in the controls, but the difference was not statistically significant (Beranek et al., 2023).

To identify hub genes regulated by DNAm as biomarkers for psoriasis, Liu et al. (2021) conducted multi-omics analysis and machine learning algorithm analysis on the gene expression and methylation datasets of PP and NN, used highly methylated genes for immune cell infiltration analysis and found that PP is mainly composed of activated dendritic cells, resting mast cells, T follicular helper cells, and other components. The study also found that hypermethylated and downregulated genes were mainly enriched in glucose homeostasis, the AMP-activated protein kinase signaling pathway, lipid storage disease, partial lipodystrophy, and insulin resistance. Insulin receptor substrate 1 (IRS1), Rho guanine nucleotide exchange factor 10 (ARHGEF10), and retinoic acid-induced 14 (RAI14) have high diagnostic accuracy and can serve as potential biomarkers for the diagnosis of psoriasis. Xing et al. (2022) obtained 767 DEGs and differentially methylated region (DMR) genes between psoriasis and healthy control samples, and further screened 33 DMEGs based on transcriptome and DNAm data. Finally, they identified the key gene *GJB2*, which is significantly overexpressed and has low methylation levels in psoriasis samples and may participate in the pathogenesis of psoriasis by disrupting the immune system, regulating the cell cycle, and disrupting the skin barrier. Therefore, *GJB2* may be a diagnostic biomarker and therapeutic target for psoriasis in the future, and the use of the GJB2 inhibitors (OCTANOL and CARBENOXOLONE) may become a new treatment strategy for psoriasis.

Although studies have focused on the transcriptome, microRNAs, and DNAm, there is a lack of comprehensive analyses that integrates omics data from the same patient sample. Laha et al. (2025) conducted a multi omics integrated analysis of RNA sequencing, microRNA sequencing, and DNAm datasets from diseased skin and adjacent normal skin of patients with psoriasis and found that genes and biological processes were regulated independently or in combination by DNAm and microRNA, and DNAm was more significant in regulating immune and inflammatory responses. Some classic pathways of psoriasis (Th17 and JAK-STAT signaling pathways) are simultaneously regulated by miRNAs and DNAm. This study revealed a complex gene regulatory network involved in the pathogenesis of psoriasis through DNAm and microRNAs.

### Substances affecting DNA methylation in the pathogenesis of psoriasis

S-Adenosine homocysteine hydrolase (AHCY), a key regulatory enzyme that maintains normal intracellular methylation reactions, has attracted the attention of researchers owing to its potential impact on DNAm in psoriasis. Liu et al. (2024a) found that the expression of *AHCY* is upregulated in psoriatic lesions and that the expression of *AHCY* was positively correlated with disease severity. Downregulation of AHCY can reduce DNAm levels, inhibit proliferation and abnormal differentiation of keratinocytes, and improve the symptoms of psoriasis. This study highlights the potential of AHCY as a promising therapeutic target for psoriasis. Similarly, researchers have noticed AHCY as a potential therapeutic target and have applied AHCY inhibitors to IMQ-induced psoriatic mice to investigate the mechanism of psoriasis treatment. Methyl 4-(adenin-9-yl)-2-hydroxybutanoate (DZ2002), a reversible AHCY inhibitor, had been reported that it was a viable treatment in murine psoriatic skin inflammation (Lin et al., 2018). Chen et al. (Sheng et al., 2021) further explored the mechanisms of DZ2002 in psoriasis and found that DZ2002 was differentially regulated by reducing the methylation of *GATA3* promoter and increasing the methylation of *LCN2* promoter, upregulating *GATA3* and inhibiting the expression of *LCN2*, thereby reducing inflammatory infiltration in psoriatic lesions.

DNA hypomethylation of the aryl hydrocarbon receptor repressor (AhRR), an epigenetic marker of environmental pollutants, causes skin disease. Um et al. (2021) confirmed that AhRR is hypomethylated and overexpressed in peripheral blood mononuclear cells and keratinocytes (HaCaT) in patients with psoriasis. After treatment with 2,3,7,8-tetrachlorodibenzo-p-dioxin (TCDD), another environmental pollutant, the expression level of AhRR in HaCaT cells increased, and it was confirmed that this increase induced pro-inflammatory cytokines in cells through the NF-κB signaling pathway, thereby promoting psoriasis-like inflammation. Therefore, inhibition of the effects of AhRR appears to be a potential therapeutic approach for psoriasis.

The mammalian target of rapamycin (mTOR) inhibitor rapamycin (RAPA) has also been reported to be involved in the pathogenesis of psoriasis (Dogan and Biray Avci, 2018; Yoon, 2017). Gao and Si (2018) found that tropomyosin (*TPM*) 1 and *TPM2* exhibited low expression and high methylation levels in psoriatic lesions, cell models, and animal models. In *in vivo* and *in vitro* experiments, RAPA has been shown to inhibit HaCaT cell proliferation in psoriasis or human epidermal keratinocyte models, reduce the methylation levels of *TPM1* and *TPM2* and restore their expression by inhibiting the ERK1/2 and mTOR signaling pathways to treat psoriasis. These studies provide new insights into the clinical application of psoriasis treatments.

### DNA methylation affect the pathological characteristics of psoriasis

DMSs enrichment in patients with psoriasis may affect their pathological characteristics of psoriasis (Figure 1). Munro microabscesses are a typical pathological feature of psoriasis and are characterized by the aggregation of neutrophils in the epidermis of psoriatic lesions. Studies have indicated that DNAm may regulate the progression of Munro microabscesses and thus affecting the course of psoriasis (Chandra et al., 2018; Xu et al., 2022). Xu et al. (2022) conducted genome-wide DNAm and differential methylation analyses and observed 647 overlapping DMSs associated with Munro microabscesses. GO analysis revealed that DNAm is likely regulates the binding of AP1 members and the recruitment of neutrophils in the epidermis via the TGF-β and Th17 pathways. Further analysis showed that DNAm regulated the expression of several genes, including *CFDP*, *SIRT6*, *SMG6*, *TRAPPC9*, *HSD17B7* and *KIAA0415*, which may contribute to the development of Munro microabscess. Chandra et al. (2018) also observed that psoriatic lesions with distinct histopathological features exhibit unique methylation profiles. For example, psoriatic skin with Munro microabscesses is enriched with DMGs involved in neutrophil chemotaxis. These findings suggest that DNAm regulates characteristic psoriatic pathologies through modulation of key gene expression, providing an epigenetic rationale for the development of specific targeted therapies.

### DNA methylation of psoriasis-related genes and other diseases

Epidemiological studies have shown that psoriasis is often accompanied by systemic diseases and constitutes an interactive relationship (Gao et al., 2021). There is a potential risk of cervical squamous cell carcinoma (CESC) in patients with psoriasis (Liu et al., 2024b). 27 key genes associated with psoriasis and CESC were identified, *NCAPH*, *UHRF1*, *CDCA2*, *CENPN* and *MELK* were identified as hub genes. Liu et al. (2024b) also found that chromosomal mitotic region segregation, nucleotide binding, and DNAm were the major enrichment pathways for common DEGs of psoriasis in the mitotic cell cycle.

DNAm has also revealed a potential link between depression and inflammatory diseases. Lapsley et al. (2020) compared DNAm analysis of salivary genes between a group of college students with a history of depression and healthy controls. It was found that patients with depression exhibited increased DNAm of *LCE* gene clusters and *MIR4520A/B*, which are immune genes associated with inflammatory diseases, such as psoriasis (Shen et al., 2015). In addition, DNAm changes in some immune genes may occur before depression, suggesting that depression and psoriasis may have potentially common pathways, and epigenetic modifications may serve as early biomarkers for depression.

## Identification and prediction of psoriatic arthritis in patients with psoriasis

Psoriatic arthritis (PsA) is a potentially deforming and disabling inflammatory arthritis that significantly increases the disease burden and severely affects the quality of life of patients with psoriasis. Early identification and prediction of PsA are critical for patient prognosis. Pollock et al. (2019) focused on the genetic risk of psoriasis and PsA, determined DMR and CpGs through DNAm analysis of sperm cells, and found that methylation of DMR in the first exon of *IL22* is associated with arthritis, which may be a germline risk locus for PsA. However, further research is needed to rule out the potential impact of endogenous and exogenous environmental factors on the genetic risk to germ cells. In another study, 883 differentially methylated positions (DMPs) affecting 548 genes in CD4^+^ T cells of psoriasis and PsA patients were identified, 69.2% of DMPs were associated with IFN related genes, and significantly enriched in the molecular functions of “cAMP dependent protein kinase inhibitor activity” and “cAMP dependent protein kinase regulator activity” (Natoli et al., 2023). The differential methylation characteristics of CD4+T cells can serve as potential molecular markers for distinguishing psoriasis from PsA. In addition, IL-17/TNF inhibitors treatment can correct the DNAm pattern of IL-17/TNF-related genes and improve the PASI score of patients with psoriasis.

Cruz-Correa et al. (2023) identified epigenetic markers that predict PsA before the onset of musculoskeletal symptoms by analyzing the whole-blood DNAm profile of patients with psoriasis. 36 highly correlated methylation sites in 15 genes (such as *FBXO27* and *ZNF385D*) were screened, and pathway analysis revealed that these sites were associated with non-classical inflammatory pathways, such as Wnt, PI3K-AKT-mTOR, and TGF-β pathways, which are highly consistent with the pathogenesis of PsA. Verma et al. (2018) focused on the epigenetic modifications of non-lesional epidermis from patients with psoriasis, identifying over 2,000 DMSs through genome-wide DNAm profiling, most of which involve the Wnt and cadherin pathways, with a large number of DMSs in the non-lesional epidermis, suggesting the presence of a pre-psoriatic state. These studies provide ideas for identifying biomarkers for psoriasis-prone skin before disease onset, which is a novel epigenetic marker for the early warning of PsA.

Deng et al. (2022) conducted a study to differentiate PsA from other autoimmune diseases based on DNAm CpG markers. Through pyrophosphate sequencing, two DMSs (cg16459382 and cg16348668) were used to distinguish PsA from psoriasis vulgaris (PsV). Based on the identified DMS, three logistic regression prediction models were established to distinguish between PsA, rheumatoid arthritis, PsV, and healthy controls (area under the curve >0.85). The PsA diagnostic model based on peripheral blood DNAm proposed in this study can serve as a non-invasive diagnostic tool for identifying DNAm markers in PsA, which is of great significance for the early identification and intervention of diseases.

## New perspectives based on DNA methylation in psoriasis

### The impact of psoriasis on biological age based on DNA methylation

The epigenetic clock is a biological age prediction tool based on DNAm patterns. It predicts an individual’s biological age by detecting the methylation levels of specific CpG sites (regions rich in cytosine and guanine dinucleotides in DNA) in the genome. Macit et al. (2024) explored the relationship between psoriasis and epigenetic DNAm clocks in a paired case-control study. There was no statistically significant difference in epigenetic age between patients with psoriasis and controls, but patients with PsA demonstrated an accelerated PhenoAge compared with the matched controls, suggesting that PsA may accelerate biological aging through a higher inflammatory load. Previous studies have shown that DNAm is associated with the regulation of psoriasis-related susceptibility gene expression (Pollock et al., 2017). Shen et al. (2018) were interested in the biological age inferred from the DNA methylome in the skin tissue of patients with psoriasis, although they observed differential methylation between PP and PN from patients with psoriasis; however, there was no significant difference in DNAm age between skin tissues from patients with psoriasis and NN, indicating that psoriasis does not directly affect the biological age and aging process.

### DNA methylation of psoriatic lesions in different body parts

Are there any differences in DNAm of the psoriatic lesions located in different parts of the body? Wu et al. (2019) conducted an interesting study and found significant location-specific DNAm differences in psoriatic lesions (315 DMSs in the back, 291 in the limbs, and 801 in the abdomen), and that differential methylation at different anatomical sites was associated with the pathological features of psoriasis. For example, the limb-specific site cg21942490, located on *HOXA9*, is associated with hyperkeratosis, whereas the DMRs in *HOXA5* (hypermethylated) and *KIAA1949* (hypomethylated) are associated with keratinocyte development and actin regulation, respectively. This study provides new insights into pathogenesis of psoriasis.

### Molecular subtype of psoriasis based on DNA methylation

Psoriasis is typically classified into four subtypes based on clinical manifestations. Zhou et al. (2018) first classified patients with psoriasis in the Chinese Han population into three subclassifications (types I, II, and III) based on DNAm data. Among them, patients with type I delayed-onset (≥40 years old) had the highest proportion, whereas type III patients had the smallest proportion of smokers. Moreover, there were significant differences in copy number variations in *IL22* among the three subtypes. This study provides a DNAm basis for the molecular subtype of psoriasis, and based on subtype characteristics, it is helpful for individualized treatment and management of patients with psoriasis.

## Conclusion and perspectives

In this review, we provide an overview of research progress on the role of DNAm in psoriasis. It is hoped that this review will further provide potential therapeutic targets and biomarkers for psoriasis. Although some potential inhibitors of DNAm have been explored, there are currently no DNAm-targeted drugs available for the clinical treatment of psoriasis. Therefore, further research on DNAm is required to develop new drugs for psoriasis treatment.
